# Factors associated with poor adherence to medication among hypertensive patients in twelve low and middle income Sub-Saharan countries

**DOI:** 10.1371/journal.pone.0219266

**Published:** 2019-07-10

**Authors:** Diane Macquart de Terline, Adama Kane, Kouadio Euloge Kramoh, Ibrahim Ali Toure, Jean Bruno Mipinda, Ibrahima Bara Diop, Carol Nhavoto, Dadhi M. Balde, Beatriz Ferreira, Martin Dèdonougbo Houenassi, Méo Stéphane Ikama, Samuel Kingue, Charles Kouam Kouam, Jean Laurent Takombe, Emmanuel Limbole, Liliane Mfeukeu Kuate, Roland N’guetta, Jean Marc Damorou, Zouwera Sesso, Abdallahi Sidy Ali, Marie-Cécile Perier, Michel Azizi, Jean Philippe Empana, Xavier Jouven, Marie Antignac

**Affiliations:** 1 Department of Pharmacy, Saint Antoine hospital, HUEP, AP-HP, Paris, France; 2 Paris Cardiovascular Research Centre, INSERM U970, European Georges Pompidou Hospital, Paris, France; 3 Paris Descartes University, Paris, France; 4 Cardiology Department, University Hospital of Aristide Le Dantec, Dakar, Senegal; 5 Institute of Cardiology of Abidjan, Abidjan, Côte d’Ivoire; 6 Internal Medicine and Cardiology Department, University Hospital of Lamorde, Niamey, Niger; 7 University hospital of Libreville, Libreville, Gabon; 8 Cardiology Department, University Hospital of Fann, Dakar, Senegal; 9 Instituto do Coração (ICOR), Maputo, Mozambique; 10 Department of Cardiology, University Hospital of Conakry, Conakry, Guinea; 11 National University hospital of Hubert K. MAGA (CNHU-HKM), Cotonou, Bénin; 12 Cardiology Department, National University Hospital of Brazzaville, Marien NGOUABI University, Brazzaville, Congo; 13 University of Yaoundé, Ministry of Public Health, Yaoundé, Cameroon; 14 Internal Medicine Department, Régional Hospital, Bafoussam, Cameroon; 15 General Hospital of Kinshasa, Kinshasa, Democratic Republic of the Congo; 16 Department of Internal Medicine of la Gombe (CMCG), Department of Internal Medicine, Ngaliema Hospital, Kinshasa; Democratic Republic of the Congo; 17 Central Hospital of Yaoundé, Yaoundé; Cameroon; 18 Cardiology department, CH Lomé, Lomé; Togo; 19 Cardiology clinics, Nouakchott, Mauritania; 20 Hypertension Unit, Assistance-Publique Hôpitaux de Paris, Hôpital Européen Georges Pompidou, Paris, France; 21 Institut National de la Santé et de la Recherche Médicale, Centre d’Investigation Clinique, Paris, France; 22 Department of Cardiology, European Georges Pompidou Hospital, AP-HP, Paris, France; Shanghai Institute of Hypertension, CHINA

## Abstract

**Introduction:**

Over the past few decades, the prevalence of hypertension has dramatically increased in Sub-Saharan Africa. Poor adherence has been identified as a major cause of failure to control hypertension. Scarce data are available in Africa.

**Aims:**

We assessed adherence to medication and identified socioeconomics, clinical and treatment factors associated with low adherence among hypertensive patients in 12 sub-Saharan African countries.

**Method:**

We conducted a cross-sectional survey in urban clinics of both low and middle income countries. Data were collected by physicians on demographics, treatment and clinical data among hypertensive patients attending the clinics. Adherence was assessed by questionnaires completed by the patients. Factors associated with low adherence were investigated using logistic regression with a random effect on countries.

**Results:**

There were 2198 individuals from 12 countries enrolled in the study. Overall, 678 (30.8%), 738 (33.6%), 782 (35.6%) participants had respectively low, medium and high adherence to antihypertensive medication. Multivariate analysis showed that the use of traditional medicine (OR: 2.28, 95%CI [1.79–2.90]) and individual wealth index (low vs. high wealth: OR: 1.86, 95%CI [1.35–2.56] and middle vs. high wealth: OR: 1.42, 95%CI [1.11–1.81]) were significantly and independently associated with poor adherence to medication. In stratified analysis, these differences in adherence to medication according to individual wealth index were observed in low-income countries (*p*<0.001) but not in middle-income countries (*p* = 0.17). In addition, 26.5% of the patients admitted having stopped their treatment due to financial reasons and this proportion was 4 fold higher in the lowest than highest wealth group (47.8% vs 11.4%) (*p*<0.001).

**Conclusion:**

This study revealed the high frequency of poor adherence in African patients and the associated factors. These findings should be useful for tailoring future programs to tackle hypertension in low income countries that are better adapted to patients, with a potential associated enhancement of their effectiveness.

## Introduction

High Blood Pressure (BP) is the worldwide leading global burden of disease risk factor; 16.5% of total deaths and 7.0% of global disability adjusted life years are attributed to high BP [1].

Due to the aging of the population and the obesity epidemic [2], high BP is increasing and despite the availability and effectiveness of multiple antihypertensive class of drugs, hypertension remains poorly controlled worldwide. Over the past few decades, hypertension prevalence has dramatically increased in developing countries [3], especially in Sub-Saharan Africa [1, 4] and tackling its burden is regarded now as a high priority task. Among various factors, poor adherence to antihypertensive medications is a major cause of failure to control hypertension [5] and a considerable proportion of CVD events (9% in Europe) could be attributed to poor adherence to antihypertensive medications [6]. Poor adherence reduces the effectiveness of drugs and represents a significant barrier to achieving better patient outcomes [7–9]. According to the WHO, the extent of poor adherence in developing countries is likely to be even higher given the lack and unequal access to health care and medications [10].

Given the scarcity of health resources available in developing countries, especially in Sub-Saharan Africa, only quality improvement interventions that are cost-efficient are likely to be feasible [11]. Interventions to improve adherence to medication should have multifaceted dimension [12] and may represent a great opportunity to improve cardiovascular outcomes and reduce health care spending [13, 14] and underutilization of already limited treatment resources [10]. However, to be effective such interventions must be tailored to the particular economical, geographical, sociological and educational context of the patients. Therefore, there is a serious need of accurately assessing not only adherence, but also to identify barriers and behaviors that influences adherence to medication.

Studies on adherence to medication are scarce in sub-Saharan Africa and usually come from single-center studies using various measures of adherence [15] and do not precisely assess other factors specific to the African context.

To cover this unmet need we performed a new analysis of the EIGHT (Evaluation of Hypertension in Sub-Saharan Africa) Study [16], a large multinational study conducted in 12 sub-Saharan African countries. In this paper, we assessed adherence to medication and identify socioeconomics, clinical and treatment factors associated with poor adherence in African hypertensive patients.

### Objectives

We therefore performed an analysis of the EIGHT study to assess adherence to medication and to identify socioeconomics, clinical and treatment factors associated with poor adherence among hypertensive patients in 12 sub-Saharan African countries.

## Methods

### Study design and setting

We conducted an observational cross sectional study during outpatient consultations in cardiology departments of 29 hospitals from 17 cities across 12 sub-Saharan African countries (Benin, Cameroon, Congo (Brazzaville), Democratic Republic of the Congo, Gabon, Guinea, Côte d’Ivoire, Mauritania, Mozambic, Niger, Senegal, Togo) between January 2014 and November 2015.

The study was conceived and designed by a multidisciplinary collaborative team of epidemiologists, cardiologists and pharmacists from Africa and France. The team had extensive prior research experience and existing collaborations with a network of physician-scientists in Africa such as in the field of rheumatic heart disease [17], sickle cell disease [18], quality of cardiovascular drugs [19, 20] which aided planning and launch of the present study.

The study was approved by the Ile-de-France III ethics committee (Number 2014-A00710-47) and was declared to the National Commission of Informatics (Number 1762715).

### Participants

Patients were enrolled during outpatient consultations in the cardiology departments of the participating hospitals. Patients with hypertension diagnosis, ≥18 years old were eligible to participate. Each patient received an information leaflet about the study. Further, the on-site physician presented and explained the study in the regional language to all patients meeting eligibility criteria and collected patient consent. Due to the observational, non-interventional nature of the study, with anonymized data, informed consent was verbally obtained and registered by the same physician. Patients who agreed to participate completed a standard questionnaire while waiting for their appointment. Participating investigators (physicians) at each center received a training note detailing the study with standardized instructions on how they should interact with the patients while filling the questionnaire in the survey.

### Questionnaire and clinical data report

#### Questionnaire

A dedicated questionnaire was conceived by the authors of the study. A first section was completed by patients and collected data on patient’s demographics (age, gender, marital status etc.) and adherence to treatment. Adherence to medication was assessed by the 8-items Medication Adherence Scale [21]. The questionnaire is scored from 0 to 8 with scores of <6, 6 to <8, and 8 reflecting low, medium, and high adherence, respectively. Additional items on patient financial situation on adherence and reasons of non-adherence (list of options asking patients to document the main reason for not taking their treatment) were included. A second section was filled out by the physician during the consultation and collected data on patient’s sociodemographic, drug regimen and clinical data.

A pilot investigation involving 90 patients who tested the questionnaire was conducted in January 2014 in Côte d’Ivoire. This pilot study confirmed patient’s understanding, feasibility of completion, and resulted in few modifications which were incorporated in the questionnaire given to patients in the final survey (S1 File Survey questionnaire).

#### Country level income and individual wealth category

The country income was obtained from the World Bank database (http://data.worldbank.org/country, accessed 12 January 2015) and categorized into low income and middle income countries. Individual patient wealth categories were assessed by the treating physician and classified as low, middle and high:

- “Low” defined poor patients who have difficulties to afford medical consultations
- “Middle” defined patients who can manage with paying medical consultations
- “High” defined patients who have no difficulties to pay medical consultations.

### Statistical methods

Continuous and categorical variables were expressed as mean (standard deviation) and numbers (percentages) where appropriate. Comparisons on qualitative variables were performed with the chi-squared test while those on quantitative variables were performed with the Student test.

First, the odds ratios (ORs) and 95% confidence intervals of socioeconomics, clinical and medication factors for low adherence were estimated in logistic regression. A random effect on the country was added (generalized estimated equation models) to account for inter-country variability. Then, in a multivariate analysis, models were adjusted on all factors with a pValue of less than 0.15 in the univariate analysis.

The level of adherence according to the individual wealth index was explored separately in low and middle income countries using a chi-squared test.

All analyses were performed through scripts developed in the R software (3.4.1 (2017-06-30)). The level of significance was set at *p*<0.05.

## Results

### Participants

The EIGHT study enrolled 2198 individuals in 12 sub-Saharan countries between January 2014 and November 2015. Patients’ characteristics are reported in Table 1. A total of 1017 patients (46.3%) were from 6 low-income countries (Benin, Democratic Republic of the Congo, Guinea, Mozambic, Niger, and Togo), and 1181 (53.7%) were from 6 middle-income countries (Cameroon, Congo [Brazzaville], Gabon, Côte d’Ivoire, Mauritania, and Senegal). A greater proportion of participants were women (60.2%). Age ranged from 19 to 95 years and mean age was 57.7±12.0 years for women and 59.2±11.4 years for men. A majority of patients were living in urban setting (n = 1702, 78.9%) compared to rural way of life (n = 455, 21.1%). Individual wealth index was low, midlevel, and high in 376 (17.6%), 1053 (49.2%), and 713 patients (33.3%), respectively. Most of the patients (n = 1816, 84.4%) were diagnosed with hypertension for more than one year prior to inclusion to the survey. Overall, 653 (29.7%), 927 (42.2%), and 543 (24.7%) of the participants were prescribed 1, 2, and ≥3 antihypertensive drugs, respectively; a quarter (24.1%) of patients admitted using traditional medicine.

**Table 1:** Characteristics of the participants according to their adherence level.

	GLOBAL	ADHERENCE TO MEDICATION			
		LOW	MEDIUM	HIGH	PValue*
N, (%)	2198 (100)	678 (30.8)	738 (33.6)	782 (35.6)	
**Age, mean (sd)**	58.3 (11.8)	58.0 (12.1)	58.4 (12.1)	58.4 (11.3)	0.81
**Male, N (%)**	874 (39.8)	258 (38.1)	299 (40.5)	317 (40.5)	0.55
**Patient wealth index, N (%)**					<0.001
Low	376 (17.5)	143 (21.7)	132 (18.4)	101 (13.2)	
Middle	1053 (49.2)	342 (51.9)	360 (50.3)	351 (45.7)	
High	713 (33.3)	174 (26.4)	224 (31.3)	315 (41.1)	
**Countries income level (low vs middle), N (%)**	1017 (46.3)	351 (51.8)	320 (43.4)	346 (44.2)	0.002
**Location (Urban vs Rural), N (%)**	1702 (78.9)	506 (76.6)	571 (78.8)	625 (81.1)	0.11
**Recent diagnosis of hypertension (<1 year), N (%)**	335 (15.6)	101 (15.1)	108 (15.0)	126 (16.5)	0.66
**Body Mass Index, mean (sd)**	27.9 (5.8)	28.0 (5.9)	27.6 (5.7)	28.1 (5.9)	0.27
**Cardiovascular risks factors, N (%)**					
None	461 (28.0)	153 (28.8)	146 (26.6)	162 (28.7)	0.77
Sedentary Lifestyle	649 (39.5)	206 (38.7)	232 (42.3)	211 (37.4)	0.23
Hypercholesterolemia	328 (19.9)	104 (19.5)	99 (18.0)	125 (22.2)	0.22
Diabetes mellitus	288 (17.5)	102 (19.2)	94 (17.1)	92 (16.3)	0.44
Hypertriglyceridemia	88 (5.3)	29 (5.5)	29 (5.3)	30 (5.3)	0.99
Tobacco use	84 (5.1)	31 (5.8)	31 (5.6)	22 (3.9)	0.27
**Cardiovascular complications (none vs a least one), N (%)**	1174 (55.8)	382 (59.0)	372 (52.3)	420 (56.5)	0.03
**Use of traditional medicine, N (%)**	512 (24.1)	213 (32.0)	166 (23.1)	133 (17.9)	<0.001
**No. of antihypertensive prescribed, mean (sd)**	1.97 (0.18)	1.97 (0.17)	1.96 (0.19)	1.97 (0.18)	0.59
**Antihypertensive drug class, N (%)**					
Calcium channel blocker	1219 (57.4)	358 (54.4)	416 (58.7)	445 (58.9)	0.17
Diuretic	1167 (55.0)	355 (54.0)	387 (54.6)	425 (56.2)	0.67
Angiotensin-converting-enzyme inhibitor	981 (46.2)	299 (45.4)	343 (48.4)	339 (44.8)	0.36
Beta-blocker	466 (22.0)	129 (19.6)	154 (21.7)	183 (24.2)	0.11
Angiotensin II receptor antagonist	321 (15.1)	85 (12.9)	103 (14.5)	133 (17.6)	0.04
**Systolic BP, mean (sd)**	149.1 (23.6)	152.4 (23.8)	150.3 (24.8)	145.0 (21.5)	<0.001
**Diastolic BP, mean (sd)**	88.4 (14.3)	90.8 (15.3)	88.3 (14.9)	86.4 (12.4)	<0.001

*p-value for a chi-squared test or t-test where appropriate.

#### Adherence to medication and factors associated with low adherence level

In the study population, 678 (30.8%), 738 (33.6%), 782 (35.6%) participants had respectively low, medium and high adherence to antihypertensive drugs.

**Fig 1:**
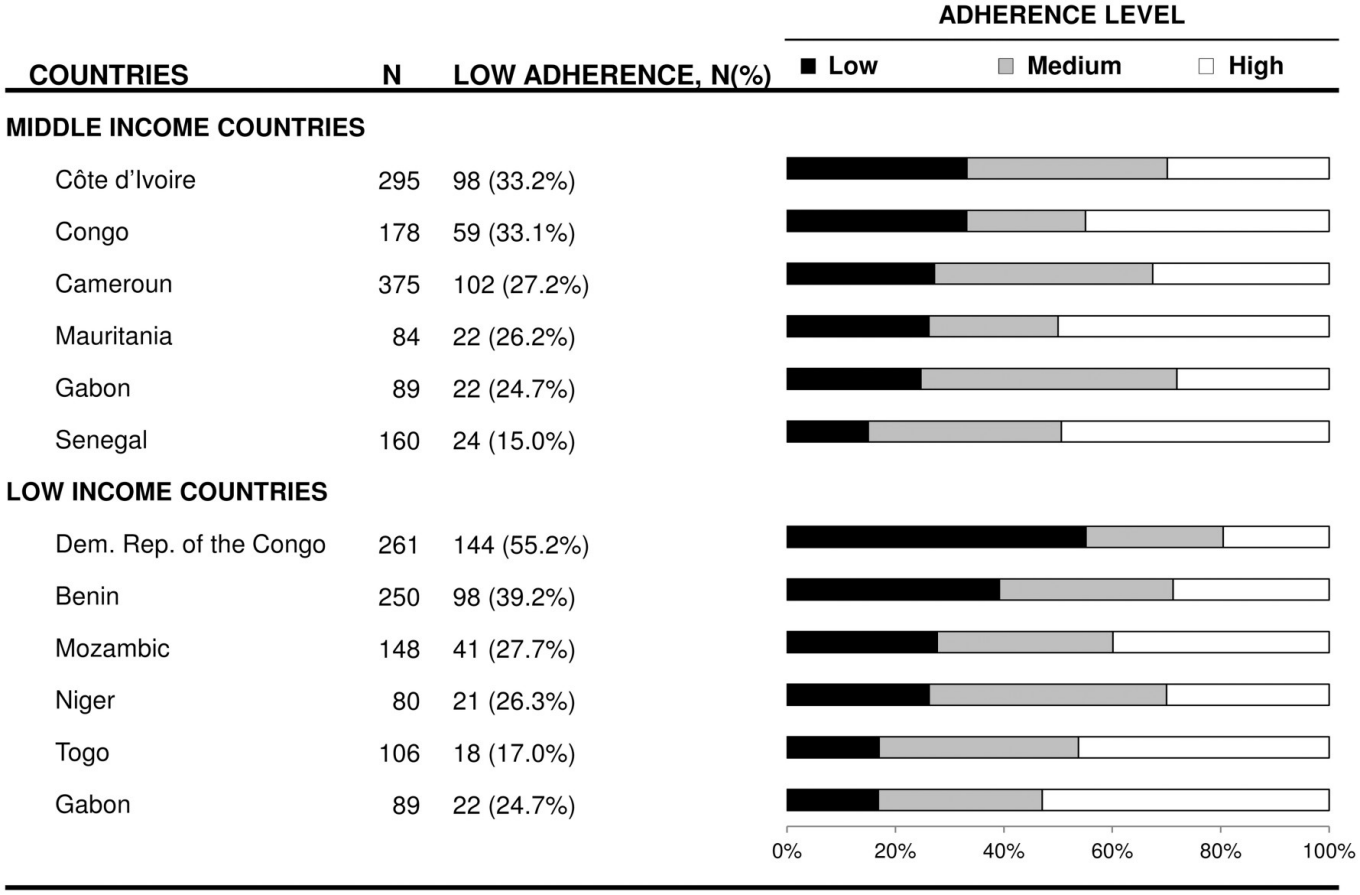
Adherence to medication according to countries. Squares represent OR and lines, 95% confidence interval (CI). ORs derived from logistic regression analysis adjusted on age, patient wealth index, complications, use of traditional medicine and antihypertensive drugs with a random effect on country to account for intra and inter-country variability.

As shown in Fig 1, the distribution of adherence level differed across countries (p<0.001), the proportion of low adherence to medication ranged from 15.0% in Senegal to 55.2% in Democratic Republic of the Congo.

In univariate analysis, low adherence was not significantly associated with age, sex, BMI, or the presence of cardiovascular complications or comorbidities (Table 2). Contrariwise, low adherence was significantly associated with patient wealth index (p<0.001); patients with a lower wealth index were more likely to be less adherent to their antihypertensive medications (OR: 1.83, 95%CI [1.38–2.45]).

**Table 2:** Odds ratios for low adherence to medication according to socioeconomics, clinical and medication factors in univariate analysis.

Outcomes	OR [95% CI]	pValue
**Age**	0.99 [0.98–1.00]	0.08
**Male**	0.95 [0.79–1.16]	0.64
**Patient wealth index**		<0.001
Low	1.83 [1.38–2.45]	
Middle	1.42 [1.14–1.80]	
High	Ref.	
**Countries income level (low vs middle)**	1.18 [0.65–2.14]	0.59
**Location (Urban vs Rural)**	0.92 [0.71–1.20]	0.51
**Recent diagnosis of hypertension (<1 year)**	0.95 [0.74–1.22]	0.56
**Body Mass Index**	1.01 [0.99–1.03]	0.17
**Cardiovascular risks factors (none vs a least one)**	0.95 [0.75–1.20]	0.67
**Complications (none vs a least one)**	0.84 [0.69–1.03]	0.10
**Use of traditional medicine**	2.22 [1.78–2.78]	<0.001
**No. of antihypertensive prescribed**	1.01[0.59–1.74]	0.96
**Antihypertensive drug class**		
Calcium channel blocker	0.77 [0.63–0.93]	0.007
Diuretic	0.98 [0.81–1.19]	0.82
Angiotensin-converting-enzyme inhibitor	1.05 [0.86–1.28]	0.65
Beta-blocker	0.92 [0.72–1.16]	0.46
Angiotensin II receptor antagonist	0.77 [0.58–1.02]	0.07

Odds ratios were estimated by logistic regression analysis with a random effect on countries using adherence (medium and high adherence) as reference category.

Patients using traditional medicine were also less adherent to their medication (p<0.001), with an OR reaching 2.22, 95%CI [1.78–2.78]. Patients prescribed a calcium channel blocker were more adherent to their treatment (OR 0.77, 95%CI [0.63–0.93] p = 0.007); this association was not observed for any other class of antihypertensive drug, nor with the number of prescribed antihypertensive drugs.

**Fig 2:**
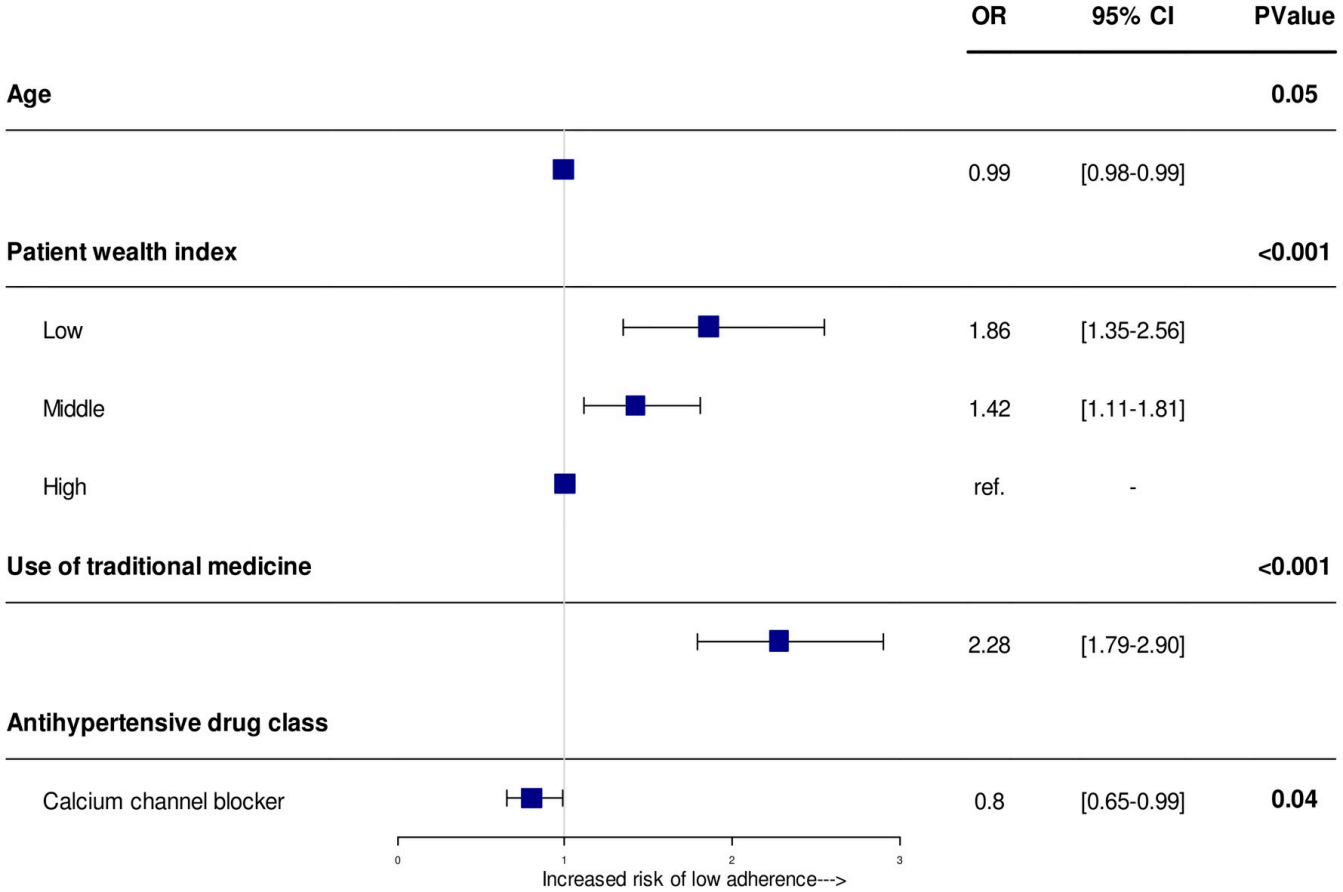
Odds ratios of patient’s factors significantly associated with low adherence level in multivariate analysis. Squares represent OR and lines, 95% confidence interval (CI). ORs derived from logistic regression analysis adjusted on age, patient wealth index, complications, use of traditional medicine and antihypertensive drugs with a random effect on country to account for intra and inter-country variability.

In the multivariate analysis, the use of traditional medicine and poor individual wealth index remained significantly and independently associated with low adherence to medication (Fig 2). The odds of low adherence increased to 2.28 fold (OR: 2.28, 95%CI [1.79–2.90], p<0.001) in patients using traditional medicine, and 1.86 fold (OR: 1.86, 95%CI [1.35–2.56]) and 1.42 (OR = 1.42, 95%CI [1.11–1.81]) in patients with low and middle individual wealth as compared to those with high individual wealth (p<0.001). Conversely, prescription of calcium channel blocker was associated with a better adherence (OR 0.8 [0.65–0.99], p = 0.007)).

### Stratified analysis by country level income

We then further investigated the association between level of adherence and individual wealth category separately in low- and middle- income countries.

**Fig 3:**
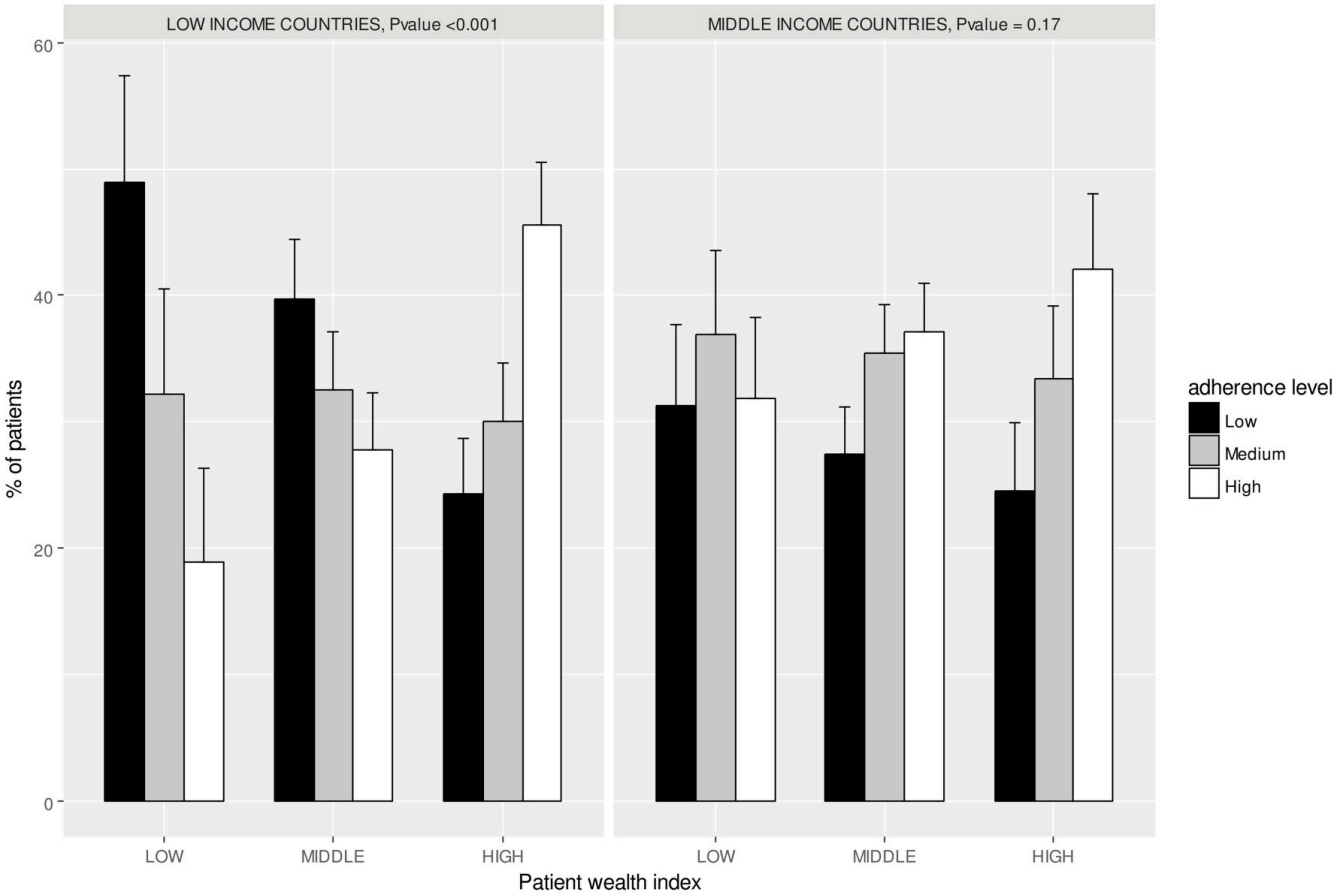
Percentage of patients according to their adherence level by patient wealth index stratified by country-level income.

In low-income countries, the proportion of low adherence increased progressively and considerably with decreasing level of individual patient wealth and was 24.3%, 39.7% and 49.0% in patients with high, middle and low individual wealth, respectively (p <0.001, Fig 3).

In contrast, in middle income countries, we observed a minor and non-significant increase of the proportion of low adherence between individual wealth categories (24.5%, 27.5% and 31.3%, in patients with high, middle and low individual wealth respectively).

#### Adherence and patients’ affordability

**Fig 4:**
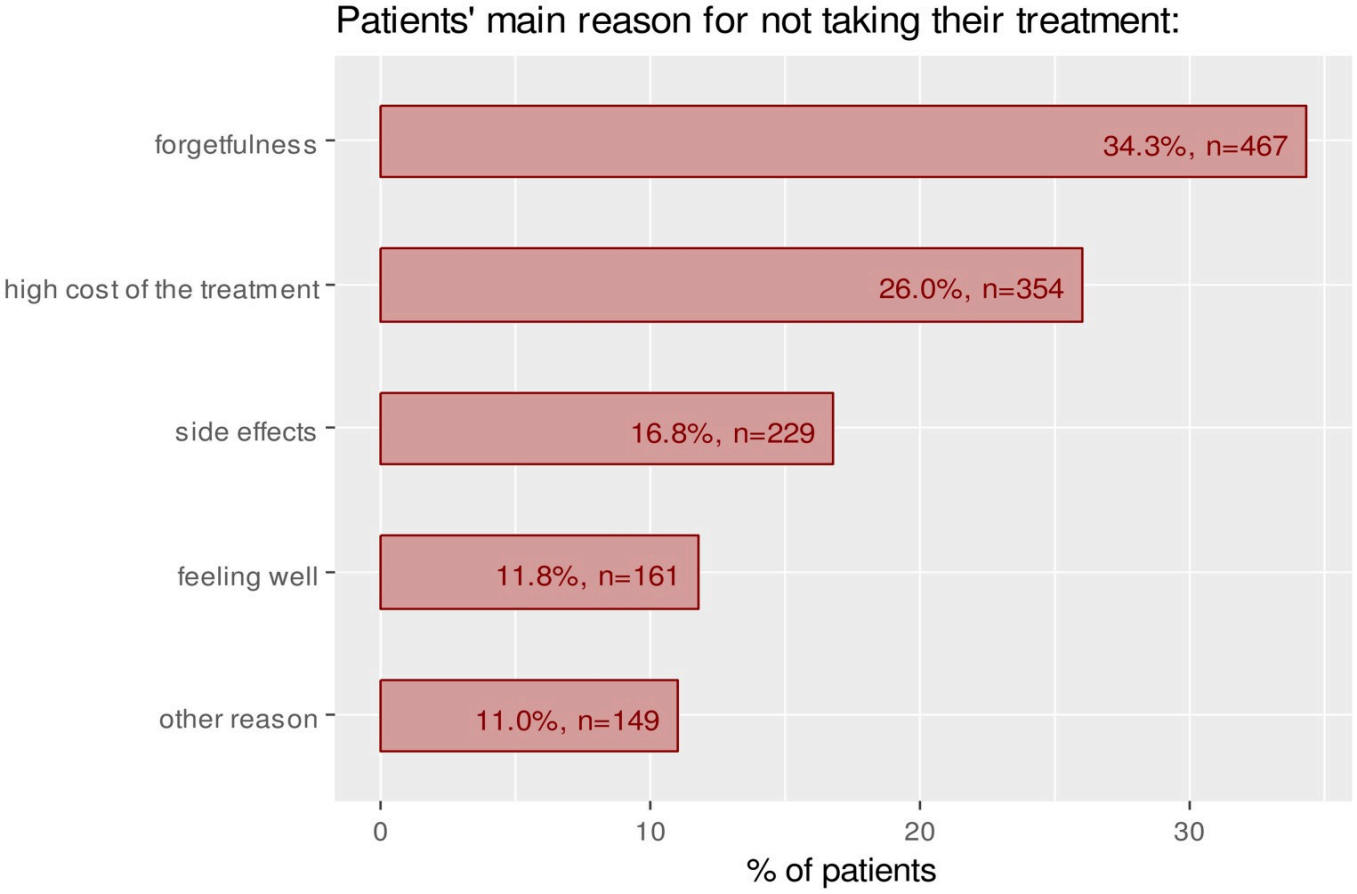
Patients’ main reason for not taking their treatment.

A total of 563 patients (26.5%) reported having stopped their treatment due to financial reasons. This proportion was much higher in the lowest wealth group (47.8%) in contrast to the highest wealth group (11.4%) (p<0.001). After forgetfulness, high cost of medications was the second main reason mentioned by patients for not taking their medications (Fig 4). Among patients categorized as good adherent, 19.2% (n = 279) reported having stopped their treatment due to financial reasons.

## Discussion

### Key results

We obtained large data on factors associated with antihypertensive drugs adherence in cardiology practices in 12 African countries. More than 60% of patients presented a suboptimal (low or medium) adherence. Furthermore, we observed several factors significantly and independently associated with low adherence including the use of traditional medicine and low wealth index.

Stratified analysis shows that these differences of adherence levels according to individual wealth index were observed in low-income countries and not in middle-income countries. More than one quarter of patients admitted having stopped their treatment due to financial reasons; this proportion was 4 fold higher in the lowest compared to the highest wealth group.

In our study, two thirds of participants had respectively low or medium adherence to antihypertensive medication. A systematic review of studies evaluating adherence to antihypertensive drugs in developing countries was published in 2017 [15]. Of the 22 studies selected, 6 were located in sub-Saharan African countries (3 in Nigeria, 1 in Ethiopia, 1 in Ghana, 1 in Zimbabwe). The percentage of poor adherence ranged from 35.4% in Ethiopia to 93.3% in Ghana. Two of the studies used the MMAS-8 self-questionnaire, reporting a very high percentage of poor adherence (92.5% [95% CI 89.3–95.8] in Nigeria and 93.3% [95% CI 90.9–95.8] in Ghana).

There is strong consensus that medication low adherence rates are higher among African patients with respect to others regions. A meta-analysis of non-adherence to antihypertensive therapy published in 2017 [22] showed major regional differences with a higher percentage of poor adherence levels among African patients (62.4%) compared to Asians (43.5%), Europeans (36.6%), and Americans (36.6%).

The multiplicity of reasons related to patient, clinician, and health system factors make poor adherence a challenging problem to address [23]. In our study, we identified relevant factors associated with low adherence, which could help to shed light on complex issue of low adherence in the particular context of the study.

Socio-economic factors are important to consider as highlighted by the present report. It has been described that low adherence and economic level of the patients are related [24]. Our study addressed for the first time the relative association of individual wealth and country level income with adherence. Although financial inequalities represent a barrier to access to treatment, the situation is potentially even more noticeable in sub-Saharan Africa. Current programs to improve access to medication in low-income and middle-income countries operate within complex health systems and reducing the wholesale price of medicines might not always or immediately translate to improved patient access [25].

Our results underlined the strong association between low adherence and the use of traditional medicine. Indeed, in developing countries, an important part of the population regularly uses traditional medicine [26, 27] define as to a set of healthcare practices that are delivered outside of the mainstream healthcare system including traditional, complementary and alternative medicine [28]. Therefore, there is a real challenge of involving traditional healers in conventional medicine. Few collaborative projects exists in sub Saharan Africa in the fields of HIV [29] and mental disorder [30] for example. Also, effort were made to conceal these two sides of medicine, in Ghana were the government began a pilot project in 17 public hospitals for integrating herbal medicine into biomedicine [31]. In conclusion, to be widely accepted, interventions and programs to improve adherence to medication in developing countries should include traditional healthcare professionals.

Channel calcium blockers are wildly used in the treatment of hypertension in black African. This antihypertensive treatment is recommended as the first-line treatment for recently diagnosed black patients [32]. Recent study has shown that channel calcium blocker associated with hydrochlorothiazide or perindopril are more effective than other dual therapies for lowering blood pressure in sub-Saharan African patients [33]. From a patient point of view, this treatment presents less inconvenient side-effects such as cough with Angiotensin-converting-enzyme inhibitor or frequent urination with diuretics.

These reasons might explain a high level of confidence in calcium channel blocker treatment by health professionals and patients, and explain the association with adherence. However this interpretation might be specific to the African context, in Europe, the order of persistence rate from the highest to the lowest is angiotensin II receptor antagonist, angiotensin converting enzyme inhibitor, channel calcium blocker, beta-blocker, and diuretic [34].

In addition to these context specific factors, our results identified reasons for treatment discontinuation commonly known by health professionals in developed countries. Indeed, the most common cause for low adherence was forgetfulness, which accounted for more than 1/3 treatment discontinuity. Therefore, intervention to enhance adherence should be based upon medication counseling, patient education or any other action to avoid forgetfulness is a way to improve adherence [35].

We acknowledged the following limitations. First, self-reported questionnaire are known to overestimate adherence. In a meta-analysis of 11 studies (n = 1684 patients) by Shi et al [36], the pooled correlation coefficient of self-report compared with electronic drug monitoring was 0.45 [95% CI, 0.34–0.56], but self-reported rates of adherence were higher than rates of adherence measured by electronic drug monitors (84% vs 75%). Furthermore, the usual adherence questionnaires were conceived in high income countries, omitting to include discontinuation of treatment due to financial reason. In the particular context of low and middle income countries, future evaluation of adherence should include questions concerning access to medication such as patient affordability [37] and accessibility to medication [38]. However, self-reported medication adherence is a practical way to measure adherence because of its low cost and potential to be easily implemented into the clinical workflow. Also, evidence showed that self-reported adherence is predictive of clinical outcomes and especially in hypertension [6, 21].

This study has also many strengths including its multisite design, with over 2,000 patients from 29 medical centers in 17 cities from 12 countries. Furthermore, this study was supported by a strong and structured collaborative multidisciplinary network. Active involvement of African cardiologists, who are familiar with the problems of this area, helped derive specific questions and analysis.

The EIGHT study embraced 12 African countries which are usually not considered in international studies, such as meta-analysis previously cited [15, 22] or the well-known PURE study providing global data on cardiovascular epidemiology. Our analysis gave the opportunity to extend knowledge on non-adherence in the African continent. However, it is important to bear in mind that these data derived from specific urban clinics likely represent the best case scenario and the magnitude of the problem in the general population with hypertension could be underestimated.”

### Conclusion

This study revealed the high level of low adherence in African patients with hypertension, and identified the associated factors specific to the regional context. These findings should be useful for tailoring future programs to tackle hypertension in low income countries, which are better adapted to patients, with a potential associated enhancement of their effectiveness.

## Supporting information

S1 File Survey questionnaire. (PDF)

S2 File Study dataset, individual data about patients’ characteristics and answers to the questionnaire used in the study. (CSV)
