# Supplementary material for: Factors associated with poor adherence to medication among hypertensive patients in twelve low and middle income Sub-Saharan countries
Source: PLoS One. 2019 Jul 10;14(7):e0219266. doi: 10.1371/journal.pone.0219266 (PMC6619761; doi:10.1371/journal.pone.0219266)
Supplement: S1 File — (PDF) [file pone.0219266.s001.pdf]

S1 File: Survey questionnaire

HYPERTENSION ACTION : EIGHT Study

EVALUATION OF ADHERENCE TO CARDIOVASCULAR TREATMENT AMONG HYPERTENSIVE PATIENTS IN AFRICA

QUESTIONNAIRE

Self-questionnaire (to be filled by the patient)

Date: _____ / __________ / __________

Last Name: ______________________________

First name: ______________________________

Address: Region: ___________________________

Village or city: ___________________________

Neighbourhood (for cities only):___________________________

Phone number: ______________________________

Gender: Male □ Female □

Nationality: _____________________________

Age: ______ years

Date of birth: _________ / ____________ / __________

Current occupation: ___________________________________

Marital status: Single □ Married □ Civil union □ Divorced □ Widow □

Number of children: ___
Number of children still living at home: ___

Dear Sir/Madam,

Auto-questionnaire regarding salt-intake

(to be filled by the patient)

If the patient is a man and his wife is with him, she can help answering the following questions.

S1. Has your Doctor asked you to reduce your salt intake ?

Yes ☐ No ☐ Unknown ☐

S2. Since you were diagnosed with hypertension, your dietary salt consumption:

Increased ☐
Remained stable ☐
Decreased slightly ☐
Decreased substantially ☐
Unknown ☐

S3. You think that your dietary salt is

Rather absent ☐
Low ☐
Normal ☐
High ☐
Unknown ☐

S4. Who makes dinner at home?

You ☐
Your spouse ☐
A cook ☐
Other (specify) ☐: ________________

S5. In average, for how many people do you cook for per meal?

__________ Unknown ☐

S6. In average, a pot is made for:

1 meal ☐
2 meals ☐
> 2 meals ☐
Unknown ☐

S7. While preparing the meal, do you add bouillon cube like Maggi®, Jumbo® or others?

☐ Yes If yes,
1 cube per pot ☐
2 cubes per pot ☐
3 cubes per pot ☐
>3 cubes per pot ☐
Unknown ☐

☐ No
☐ Unknown

S8. While preparing the meal, do you add salt in the pot (or in the cooking water)?

Yes ☐ No ☐ Unknown ☐

If yes,
1 pinch (or tablespoon) of salt in average per pot ☐
2 pinches (or tablespoon) of salt in average per pot ☐
3 pinches (or tablespoon) of salt in average per pot ☐
Over 3 pinches (or tablespoon) of salt in average per pot ☐
Unknown ☐

S9. How many times do you have salted fish per week?

Never ☐
Less than 1/ week ☐
Twice a week ☐
Every day ☐
Many times per day ☐
Unknown ☐

S10. How many dried fish do you buy each day?
___________Francs CFA in numbers: _______ fishes Unknown ☐

S11. How many bouillon cube like Maggi®, Jumbo® or other, do you buy each day?
___________Francs CFA in numbers: ________ cubes Unknown ☐

S12. Do you add salt to you served meal?

- Never ☐
- Seldom ☐
- Often ☐
- Always ☐
- Unknown ☐

S13. Do you add bouillon cubes, flavors or sauces (like Maggi®, Jumbo® …) directly on your plate?

- Never ☐
- Seldom ☐
- Often ☐
- Always ☐
- Unknown ☐

S14. Do you eat salted peanuts?

- Never ☐
- Seldom ☐
- Often ☐
- Always ☐
- Unknown ☐

Auto-questionnaire regarding treatment
(to be filled by the patient)
If you are receiving any medication for hypertension (high blood pressure), please answer the questions below by ticking a box for each question:

T1. Do you have high blood pressure (hypertension)
Yes ☐ No ☐

T2. How many pills do you take every day?
1 ☐ 2 ☐ 3 ☐ 4 ☐ 5 ☐ 6 ☐ 7 ☐ 8 ☐ 9 ☐ 10 ☐ >10 ☐

T3. Do you ever use traditional medicine as a treatment for your cardiovascular disease ?
Yes ☐ No ☐

T4. Do you have other treatments besides your treatment for your cardiovascular disease?
Yes ☐ No ☐

T5. Do you sometimes forget to take your pills?
Yes ☐ No ☐

T6. People sometimes miss taking their medications for reasons other than forgetting. Thinking over the past two weeks, were there any days when you did not take your medicine?
Yes ☐ No ☐

T7. Have you ever cut back or stopped taking your medication without telling your doctor, because you felt worse when you took it?
Yes ☐ No ☐

T8. When you travel or leave home, do you sometimes forget to bring along your medication?
Yes ☐ No ☐

T9. Did you take your medicine yesterday?
Yes ☐ No ☐

T10. When you feel like your health concern is under control, do you sometimes stop taking your medicine? Yes ☐ No ☐

T11. Taking medication everyday is a real inconvenience for some people. Do you ever feel hassled about sticking to your treatment plan?
Yes ☐ No ☐

T12. How often do you have difficulty remembering to take all your medications?
Never/Rarely ☐
Once in a while ☐
Sometimes ☐
Usually ☐
All the time ☐

T13. Some medications are expensive. Did you ever skip taking your treatment for financial reasons?

Yes ☐ No ☐

T14. Did you ever switch your medications to less expensive ones?

Yes ☐ No ☐

T15. Can you identify the main reason for not taking your treatment?

- Its side effects ☐
- High cost of the treatment ☐
- Inconvenient treatment schedule ☐
- The high number of pills to be taken daily ☐
- Forgetfulness ☐
- The embarrassment of taking the pills in public ☐
- Feeling well ☐
- Other: please specify: ____________________________ ☐

T16. Can you identify the second main reason for not taking your treatment?

- Its side effects ☐
- High cost of the treatment ☐
- Inconvenient treatment schedule ☐
- The high number of pills to be taken daily ☐
- Forgetfulness ☐
- The embarrassment of taking the pills in public ☐
- Feeling well ☐
- Other: please specify: ____________________________ ☐

T17. Where do you get your treatment from ?

☐ Pharmacy ☐ Market ☐Other: _____________

Questionnaire for the patient's physician:

Dr ______________________________

Hospital: ______________________________

City: ____________________ Country: __________________

Date: _____ / __________ / __________

Name of the patient: ______________________________

D1. Is the patient hypertensive ?

Yes ☐ No ☐

D2. According to you, what is the socio-economic category of your patient?

- Low ☐
- Medium ☐
- High ☐

D3. According to you, what is the educational level of your patient?

- No schooling ☐
- Primary ☐
- Secondary ☐
- Higher ☐

D4. Your patient lives in a:

- Urban area ☐
- Semi rural area ☐
- Rural area ☐

D5. The cardiovascular cause of the consultation is:

- Hypertension ☐
- Heart failure (HF) ☐
- Rhythm disturbance ☐
- Valvular heart disease ☐
- Coronary heart disease ☐
- Other: ______________________ ☐

D6. Your patient has a family history of

- Hypertension ☐
- Stroke ☐
- Paralysis ☐
- Diabetes ☐
- Other: ______________________ ☐

D7. What are your patient's cardiovascular risk factors?

- Active smoker ☐
- Diabetes ☐
- Hypercholesterolemia ☐
- High triglycerides ☐
- Obesity ☐
- Sedentary lifestyle ☐
- None ☐
- Other : ___________________________ ☐

D8. Does your patient have a severe disease, other than cardiovascular?

Yes □ No □

If yes, please specify:

Cancer ☐
Haematology ☐
Endocrinology/metabolism ☐
Gastroenterology/hepatology ☐
Infectious Diseases/Viral ☐
Ophthalmology/ENT ☐
Orthopaedics ☐
Psychiatry/Neurology ☐
Rheumatology ☐

D9. If the patient has hypertension, how long has it lasted for ?

< 1 year ☐ 1 to 5 years ☐ 6 to 9 years ☐ > 10 years ☐

D10. If the patient has hypertension, has he/she presented with complications?

Yes □ No □

If yes,	Mild	Severe
Ocular	☐	☐
Renal	☐	☐
Cardiac	☐	☐
Neurological	☐	☐

D11. If the patient has hypertension, what is his/her current treatment (multiple choices possible if polytherapy) :

Diuretics ☐
Beta-blocker ☐
Calcium blocker ☐
Central antihypertensive ☐
Vasodilator ☐
Angiotensin II agonists ☐
ACE inhibitors ☐

D12. Does your patient take at least one combination of drugs?

Yes □ No □

D13. Does your patient take a generic drug ?

Yes□ No □

D14. Measurements to be made during the consultation

Weight : ___________ kg

Height (measured or declared): ___________ cm

Heart rate: ___________ bpm

	1st measurement	*2nd measurement*
Systolic blood pressure (SBP):	________mmHg	________mmHg
Diastolic blood pressure (DBP):	________mmHg	________mmHg

Waist circumference (over the iliac crest) *[if possible]*: __________ cm

D15. Did the patient get help to fill in this questionnaire?

No □ Yes, by the nurse □ Yes, by the Doctor □

ACTION HYPERTENSION : Etude EIGHT

EVALUATION DE L'OBSERVANCE DANS LES PATHOLOGIES CARDIOVASCULAIRES EN AFRIQUE EVALUATION OF ADHERENCE TO CARDIOVASCULAR TREATMENT AMONG HYPERTENSIVE PATIENTS IN AFRICA

QUESTIONNAIRE

Auto-questionnaire (à compléter par le patient)

Date : ____ / ________ / ________

NOM : ________________________

Prénom : ________________________

Adresse : Région : ________________________

Village ou ville: ________________________

Quartier (pour les villes) : ________________________

N° de téléphone : ________________________

Sexe : Homme □ Femme □

Nationalité : ________________________

Age : ______ ans

Date de naissance : ________ / __________ / ________

Profession : ____________________________

Situation familiale :
célibataire □ marié(e) □ vie maritale □ divorcé(e) □ veuf(ve) □

Nombre d'enfants : ___
Nombre d'enfants encore au domicile familial : ___

<u>Auto-questionnaire concernant les habitudes de consommation en sel</u>
(à compléter par le patient):
Si le patient est un homme accompagné de son épouse, elle pourra aider à répondre aux questions suivantes.

S1. Votre médecin vous-a-t-il demandé de diminuer votre consommation en sel ?
Oui ☐ Non ☐ Ne sait pas ☐

S2. Depuis le diagnostic de votre maladie cardiovasculaire, votre consommation en sel (dans l'alimentation) :
A augmenté ☐
N'a pas changé ☐
A un peu diminué ☐
A beaucoup diminué ☐
Ne sait pas ☐

S3. Vous trouvez que votre alimentation est
Non salée ☐
Peu salée ☐
Normalement salée ☐
Très salée ☐
Ne sait pas ☐

S4. Qui prépare le dîner chez vous ?
Vous ☐
votre conjoint(e) ☐
un(e) cuisinier ou une servante ☐
autre à préciser ☐: ________________

S5. En moyenne, pour combien de personnes le repas est-il préparé ?
___________ Ne sait pas ☐

S6. En moyenne, une marmite est préparée pour
1 repas ☐
2 repas ☐
> 2 repas ☐
Ne sait pas ☐

S7. Lors de la préparation des plats, des cubes type MAGGI®, JUMBO® ou autres sont-ils ajoutés ?

☐ Oui, préciser 1 cube par marmite ☐
2 cubes par marmite ☐
3 cubes par marmite ☐
>3 cubes par marmite ☐
Ne sait pas ☐

☐ Non
☐ Ne sait pas

S8. Lors de la préparation des plats, du sel est-il ajouté dans votre marmite (ou dans l'eau de cuisson) ?

Oui ☐ Non ☐ Ne sait pas ☐

Si oui,
- 1 poignée (ou cuillère) de sel en moyenne par marmite ☐
- 2 poignées (ou cuillères) de sel en moyenne par marmite ☐
- 3 poignées (ou cuillères) de sel en moyenne par marmite ☐
- >3 poignées (ou cuillères) de sel en moyenne par marmite ☐
- Ne sait pas ☐

S9. Combien de fois par semaine mangez-vous du poisson séché ?
- Jamais ☐
- Moins de 1 fois par semaine ☐
- 2 fois par semaine ☐
- Tous les jours ☐
- Plusieurs fois par jour ☐
- Ne sait pas ☐

S10. Combien achetez-vous de poisson séché par jour ?
___________Francs CFA en nombre : _______ poissons Ne sait pas ☐

S11. Combien achetez-vous de cubes MAGGI®, JUMBO® ou autres par jour ?
___________Francs CFA en nombre : ________ cubes Ne sait pas ☐

S12. Ajoutez-vous du sel dans les aliments ou plats présents dans votre assiette ?
- Jamais ☐
- Rarement ☐
- Souvent ☐
- Toujours ☐
- Ne sait pas ☐

S13. Ajoutez-vous des flacons d'arôme ou de sauce MAGGI®, JUMBO® ou autres, dans votre assiette ?
- Jamais ☐
- Rarement ☐
- Souvent ☐
- Toujours ☐
- Ne sait pas ☐

S14. Consommez-vous des cacahuètes salées ou des arachides salés ?
- Jamais ☐
- Rarement ☐
- Souvent ☐
- Tous les jours ☐
- Ne sait pas ☐

<u>Auto-questionnaire concernant les traitements</u>
(à compléter par le patient):

Si vous recevez un traitement médicamenteux, merci de répondre aux questions suivantes, cocher une seule case par question :

T1. Etes-vous atteint d'hypertension artérielle ?

Oui ☐ Non ☐

T2. Combien de comprimés (ou gélules) différents prenez-vous chaque jour :

1 ☐ 2 ☐ 3 ☐ 4 ☐ 5 ☐ 6 ☐ 7 ☐ 8 ☐ 9 ☐ 10 ☐ >10 ☐

T3. Avez-vous parfois recours à la médecine traditionnelle pour votre maladie cardiovasculaire ?

Oui ☐ Non ☐

T4. En dehors des traitements pour votre maladie cardio-vasculaire, prenez-vous d'autres médicaments ?

Oui ☐ Non ☐

T5. Vous arrive-t-il quelquefois d'oublier de prendre vos médicaments ?

Oui ☐ Non ☐

T6. Il arrive que des personnes ne prennent pas leurs médicaments pour des raisons autres que l'oubli. Pensez aux deux dernières semaines, vous est-il arrivé au moins un jour de ne pas prendre vos médicaments ? Oui ☐ Non ☐

T7. Vous est-il déjà arrivé de diminuer ou d'arrêter de prendre votre traitement sans le dire à votre médecin, parce que vous vous sentiez plus mal lorsque vous le preniez ?

Oui ☐ Non ☐

T8. Lorsque vous voyagez ou quittez votre domicile, vous arrive-til d'oublier d'emporter avec vous vos médicaments ? Oui ☐ Non

T9. Avez-vous pris vos médicaments hier ?

Oui ☐ Non ☐

T10. Lorsque vous sentez que votre problème de santé est maîtrisé, vous arrive-t-il de stopper votre traitement ? Oui ☐ Non ☐

T11. Prendre son traitement tous les jours constitue une vraie gêne pour certains patients. Vous arrive – t – il quelquefois de ne pas supporter de prendre votre traitement ?

Oui ☐ Non ☐

T12. Combien de fois avez-vous eu des difficultés à vous rappeler de prendre votre traitement ?

Jamais/ rarement ☐

Une fois ☐

Parfois ☐

Souvent ☐

Tout le temps ☐

T13. Certains médicaments sont chers. Vous est-il arrivé de ne pas prendre votre traitement pour des raisons financières ? Oui ☐ Non ☐

T14. Vous est-il arrivé de changer vos médicaments pour des médicaments moins chers ?
Oui ☐ Non ☐

T15. Cocher votre principale raison de ne pas prendre votre traitement

- les effets indésirables ☐
- le coût élevé des traitements ☐
- les horaires de prise contraignants ☐
- le nombre important de médicaments chaque jour ☐
- les oublis ☐
- la gêne à prendre mes médicaments devant les autres ☐
- lorsque je me sens bien ☐
- autre : à détailler : ______________________________ ☐

T16. Cocher la 2ème raison de ne pas prendre votre traitement

- les effets indésirables ☐
- le coût élevé des traitements ☐
- les horaires de prise contraignants ☐
- le nombre important de médicaments chaque jour ☐
- les oublis ☐
- la gêne à prendre mes médicaments devant les autres ☐
- lorsque je me sens bien ☐
- autre : à détailler : ______________________________ ☐

T17. Où vous approvisionnez vous en médicament ? (plusieurs réponses possibles)

☐ A la pharmacie ☐ sur le marché ☐autre : ______________

Questionnaire destiné au Médecin prenant en charge le patient

Dr ______________________________

Hôpital : ______________________________

Ville : ____________________ Pays : __________________

Date : _____ / __________ / __________

Nom du patient : ______________________________

D1. Le patient est-il atteint d'hypertension artérielle ?

OUI ☐ NON ☐

D2. Selon vous, à quelle catégorie socio-économique appartient votre patient ?

- Bas ☐
- Moyen ☐
- Elevée ☐

D3. Selon vous, quel est le niveau d'étude de votre patient ?

- Non scolarisé ☐
- Iaire ☐
- IIaire ☐
- Supérieur ☐

D4. Votre patient habite en milieu

- Urbain ☐
- Semi rural ☐
- rural ☐

D5. La cause cardiovasculaire de la consultation est (plusieurs réponses possibles)

- Hypertension artérielle (HTA) ☐
- Insuffisance cardiaque (IC) ☐
- Trouble du rythme ☐
- Valvulopathie ☐
- Coronaropathie ☐
- Autre : ____________________________ ☐

D6. Votre patient présente des antécédents familiaux

- d'hypertension artérielle ☐
- d'accidents vasculaires cérébraux ☐
- de paralysie ☐
- de diabète ☐
- Aucun ☐
- Autre : ____________________________ ☐

D7. Votre patient présente des facteurs de risque cardiovasculaires

- Tabagisme actif ☐
- Diabète ☐
- Hypercholestérolémie ☐
- Hypertriglycéridémie ☐
- Obésité ☐
- Sédentarité ☐
- Aucun ☐
- Autre : ____________________________ ☐

D8. Votre patient présente-t-il une pathologie sévère autre que cardiovasculaire ?
Oui □ Non □
Si oui, préciser le type de pathologie :

Cancérologie ☐
Hématologie ☐
Endocrinologie / métabolisme ☐
Gastro-entérologie / hépatologie ☐
Infectiologie/virologie ☐
Ophtalmologie/ORL ☐
Orthopédie ☐
Psychiatrie/neurologie ☐
Rhumatologie ☐

D9. Si votre patient est atteint d'hypertension, depuis quand est-elle connue
< 1 an ☐ 1 an à 5 ans ☐ 6 à 9 ans ☐ > 10 ans ☐

D10. Si votre patient est atteint d'hypertension, a-t-il présenté des complications ?
Oui □ Non □

Si oui,	Débutante	Sévère
Oculaire	☐	☐
Rénale	☐	☐
Cardiaque	☐	☐
Cérébrale	☐	☐

D11. Si votre patient est atteint d'hypertension, quels sont ses traitements pour l'hypertension (en cas d'association, cocher plusieurs cases):
Diurétique ☐
Bétabloquant ☐
Inhibiteur calcique ☐
Antihypertenseur central ☐
Vasodilatateur ☐
Antagoniste de l'angiotensine II (ARA II) ☐
Inhibiteur de l'enzyme de conversion (IEC) ☐

D12. Votre patient prend t-il au moins une association fixe de traitement ?
Oui □ Non □

D13. Votre patient prend t-il au moins un générique ?
Oui □ Non □

D14. Mesures à réaliser au cours de la consultation

Poids du patient : __________kg
Taille (mesurée ou déclarée): __________cm
Fréquence cardiaque : ___________

	1ère mesure	*2ème mesure*
Pression artérielle systolique (PAS) :	_________mmHg	_________mmHg
Pression artérielle diastolique (PAD):	_________mmHg	_________mmHg

Périmètre abdominal (au niveau des crêtes iliaques) *[si possible]* : ___________ cm

D15. Le patient a-t-il été aidé pour compléter l'auto-questionnaire ?
Non □ Oui, par l'infirmier (ère) □ Oui, par le médecin □
